# ﻿New *Piper* species from the eastern slopes of the Andes in northern South America

**DOI:** 10.3897/phytokeys.206.75971

**Published:** 2022-08-26

**Authors:** William Trujillo, Edwin Trujillo Trujillo, Fausto Andrés Ortiz-Morea, Diego A. Toro, M. Alejandra Jaramillo

**Affiliations:** 1 Grupo Investigaciones territoriales para el uso y conservación de la biodiversidad. Fundación La Palmita, Centro de Investigación. Cra 4 # 58–59 piso 2, Bogotá, Colombia Grupo Investigaciones territoriales para el uso y conservación de la biodiversidad. Fundación La Palmita Bogotá Colombia; 2 Grupo de Investigación en Agroecosistemas y Conservación en Bosques Amazónicos - GAIA, Facultad de Ingeniería, Universidad de la Amazonia, Cl. 17 Diagonal 17 con Cra. 3F, 180 002, Florencia, Caquetá, Colombia Universidad de la Amazonia Florencia Colombia; 3 Grupo DIVERSITAS, Facultad de Ciencias Básicas y Aplicadas, Universidad Militar Nueva Granada, km 2 Vía Cajicá–Zipaquirá, Cajicá, Colombia Universidad Militar Nueva Granada Bogotá Colombia

**Keywords:** Colombia, Ecuador, north-western Amazon, Peru, *Piperhoyoscardozii*, *Piperindiwasii*, *Pipernokaidoyitau*, *Pipervelae*, Piperaceae

## Abstract

We describe four new species of *Piper* from the Amazonian slopes of the northern Andes. *Piperhoyoscardozii* is distinguished from similar climbing species, *P.dryadum* and *P.flagellicuspe*, by its longer peduncles. The Amazonian species *Piperindiwasii* is distinguished from *P.scutilimbum* from Panama and northern Colombia by the narrowly spatulate leaf base extension. *Pipernokaidoyitau* is characterised by the presence of larger leaves and longer spikes than similar species, *P.anonifolium* and *P.hostmannianum*. Finally, *P.velae* is characterised by cordulate leaf bases in all nodes, petioles 0.8–1.5 cm long and pubescent fruits, which easily distinguish it from the related species, *P.holdridgeanum*.

## ﻿Introduction

*Piper* L. is the most diverse and representative genus in Piperaceae, encompassing ca. 2600 species (Callejas-Posada 2020). *Piper* represents an extraordinary diversification amongst early diverging angiosperms. It is a Pantropical group (Jaramillo and Manos 2001) and its highest diversity lies in the Neotropics (ca. 1800 species; *fide*Ulloa-Ulloa et al. 2018 onwards). The growth forms of the genus are very variable and are most commonly perennial shrubs and suffrutices, growing mainly in the understorey of rainforests. Additionally, other *Piper* species are lianas, rarely caulescent herbs, hemi-epiphytes or trees not taller than 15 m (Callejas-Posada 2020). *Piper* is characterised by simple, alternate leaves and jointed stems with enlarged nodes; many species produce pearl bodies on the leaves or stems (Tepe et al. 2004), but the most distinctive morphological feature of the genus is the production of solitary spikes, upright or pendant, which contain dozens to thousands of apetalous flowers. Many *Piper* species are rich in essential oils, which can be found in many tissues and organs, including the fruits, seeds, leaves, branches, roots and stems (Salehi et al. 2019). Although very few species have significant global economic value, many are locally important for their use by native populations for medicinal and culinary purposes (Colvard et al. 2006; Cáceres and Kato 2014).

*Piper* has an extensive history of taxonomic and nomenclatural instability. The leading causes for that uncertainty are the challenges of interpreting morphological characteristics and the limited geopolitical circumscription of taxonomists’ work (Ramírez-Amezcua 2016; Callejas-Posada 2020). *Piper* flowers are small and morphologically homogeneous and the number of stamens and their position, characters of diagnostic importance, are challenging to see or interpret. Not all stamens develop at anthesis (Tucker 1982a, b) and the anthers, articulated at the filaments, fall off quickly after anthesis (Valentin-Silva et al. 2018). Many *Piper* species present foliar dimorphism and many specimens do not include leaves of both monopodial and sympodial axes. Description of new species, based on incomplete specimens, has led to the publication of superfluous names that later need to be submerged in synonymy (Ramírez-Amezcua 2016). Furthermore, because floristic treatments are often limited to specific countries, many species are given several names across borders (Ramírez-Amezcua 2016). Extensive fieldwork and comparisons with collections (including digitised collections) are essential to improve the taxonomy of this species-rich genera.

Observing species in the field is imperative to ensure detailed descriptions and complete specimens that support an excellent *Piper* taxonomy. Numerous essential characters cannot be included or are lost in dry specimens. Fortunately, in the last decade, W. Trujillo has collected and studied more than one thousand (1000) specimens from the Amazonian slopes of the Colombian Andes. Some of them have become type specimens of recently-described taxa (Trujillo and Callejas 2015; Trujillo and Jaramillo 2019, 2021). Continued work by the authors will bring to light more diversity in the future.

The present contribution describes four new *Piper* species from the Amazonian slopes of the Andes in Colombia, Ecuador and Peru. We use the nuclear ribosomal internal transcribed spacer (ITS) to determine their phylogenetic relationships within *Piper*. We increase to 419 the number of *Piper* species known from Colombia (Bernal et al. 2015).

## ﻿Materials and methods

Fieldwork was conducted along the Amazonian slopes of the Andes in southern Colombia, in the Department of Caquetá, during 2010–2020. We collected silica gel-dried leaf tissue samples for DNA extractions. Detailed observations in the field and examination of available herbarium collections were used to describe growth habits and phenological stages accurately. We deposited botanical specimens in COAH, COL, HUAZ and HUA (acronyms according to Thiers 2019). Detailed comparisons with morphologically similar species allowed us to recognise the four new species. Besides reviewing the literature (Trelease 1936; Trelease and Yuncker 1950; Brako and Zarucchi 1993; Jørgensen and León-Yánez 1999; Jørgensen et al. 2014; Jardim Botânico do Rio de Janeiro 2020), we examined specimens through visits to Herbaria COAH, COL, HUA, HUAZ, GH, NY, PMA, RSA and US and digitised plant specimens available on the web (e.g. JSTOR Global Plants, https://plants.jstor.org/). Measurements included here were taken from specimens collected in Colombia. To describe leaf architecture, we used terminology proposed in the Manual of Leaf Architecture (Ellis et al. 2009). To assess conservation status, we calculated area of occupancy (AOO) and extent of occurrence (EOO) using R and the package ConR (Dauby et al. 2017).

We extracted DNA from all the new species (Table 1) using a CTAB method (Doyle and Doyle 1987). We amplified the nuclear ribosomal internal transcribed spacer (ITS) according to Jaramillo and Manos (2001) and aligned sequences using previous alignments as a guide (Jaramillo et al. 2008). First, we included the new sequences in our 900+ *Piper*ITS alignment, to determine the relationships of the new *Piper* species. This preliminary analysis (not shown) served to select representatives of each major clade of *Piper* (Jaramillo et al. 2008) for the analysis presented here. Forty-two species of *Piper*, 35 from the Neotropics and seven from Asia were selected to provide comparisons with the new taxa. Maximum Likelihood (ML) phylogenetic and bootstrap (100 replicates) analyses were conducted using RAxML (Stamatakis 2014) using species from the Asian tropics to root the phylogeny.

**Table 1.** GenBank accessions for new species and reference taxa. Other GenBank accessions are available in original manuscripts (Jaramillo et al. 2008). New species in bold.

Taxon	Voucher	GenBank Accession ITS	Collection *	Publication
*Piperabalienatum* Trel.	MAJ 552	EU581075	DUKE	Jaramillo et al. (2008)
*Piperabbreviatum* Opiz	MAJ 203	EU581076	DUKE	Jaramillo et al. (2008)
*Piperaduncum* L.	MAJ 76	AF275157	DUKE	Jaramillo & Manos (2001)
*Piperalatabaccum* Trel. & Yunck.	AL 1177	KJ930372	INPA	Molina-Henao et al. (2016)
*Piperalbispicum* C. DC.	MAJ 388	AY572317	DUKE	Jaramillo & Callejas (2004)
*Piperalbozonatum* C. DC.	MAJ 697	AY326195	DUKE	Jaramillo & Callejas (2004)
*Piperamalago* L.	MAJ 561	AF215186	DUKE	Jaramillo & Manos (2001)
*Piperamplum* Kunth	MAJ 804	EU581096	RB	Jaramillo et al. (2008)
*Piperanonifolium* Kunth	AL 1242	EU581084	MG	Jaramillo et al. (2008)
	EJT 527	EU581101	MU	Jaramillo et al. (2008)
*Piperarboretum* Aubl.	MN 1565	EU581106	RB	Jaramillo et al. (2008)
*Piperbiolleyi* C DC.	CD 10896	EU581128	SRP	Jaramillo et al. (2008)
*Piperbrachypodon* (Benth.) C. DC.	MAJ 757	AY326198	DUKE	Jaramillo & Callejas (2004)
*Piperbreviamentum* C.DC.	MAJ 221	EU581134	DUKE	Jaramillo et al. (2008)
*Pipercallosum* Ruiz & Pav.	MJK 161	EU581142	SPF	Jaramillo et al. (2008)
*Pipercandollei* Sodiro	EJT 1449	EF056237	MU	Jaramillo et al. (2008)
*Pipercapense* L.	CD 11004	EU581143	SRP	Jaramillo et al. (2008)
*Pipercavendishoides* Trel. & Yunck.	MAJ 70	AF275153	DUKE	Jaramillo & Manos (2001)
*Pipercinereum* C. DC.	MAJ 66	AF275190	DUKE	Jaramillo & Manos (2001)
*Pipercocornarum* Trel. & Yunck.	RC 12493	AY326203	HUA	Jaramillo & Callejas (2004)
*Pipercordulatum* C. DC.	BCI-EL	EU581167		Jaramillo et al. (2008)
*Piperdarienense* C. DC.	MAJ 103	AF275181	DUKE	Jaramillo & Manos (2001)
*Piperdilatatum* Rich.	MAJ 858	EU581179	RB	Jaramillo et al. (2008)
*Piperdryadum* C. DC.	EJT 1047	EU581186	MU	Jaramillo et al. (2008)
*Piperexcelsum* G. Forst.	N. V.	EF635476		Jaramillo et al. (2008)
*Piperfilistilum* C. DC.	MAJ 157	AF275155	DUKE	Jaramillo & Manos (2001)
*Piperflagellicuspe* Trel. & Yunck.	MAJ 65	AF275154	DUKE	Jaramillo & Manos (2001)
*Piperhartwegianum* (Benth.) C. DC.	MAJ 781	AY326207	DUKE	Jaramillo & Callejas (2004)
*Piperhispidum* Sw.	MCS 304	EU581241	RB	Jaramillo et al. (2008)
*Piperholdridgeanum* W. C. Burger	CD 10865	EU581247	SRP	Jaramillo et al. (2008)
	JF 9128	EU581248	MO	Jaramillo et al. (2008)
*Piperhostmanianum* C. DC.	SM 25228	EU581251	NY	Jaramillo et al. (2008)
	EJT 573	EU581249	MU	Jaramillo et al. (2008)
***Piperhoyoscardozii***	**WT 4127**	**OK235346**	**UMNG**	**This study**
	**WT 4004**	**OK235347**	**UMNG**	
	**WT 4099**	**OK235348**	**UMNG**	
*Piperimberbe* Trel. & Yunck.	AB 983	EU581255	SEMO	Jaramillo et al. (2008)
***Piperindiwasii***	**ET 7254**	**OK235342**	**JBB**	**This study**
	EJT 1438	EU581390	MU	Jaramillo et al. (2008)
*Pipermaxonii* C. DC.	EJT 370	EF056270	MU	Jaramillo et al. (2008)
*Pipermultiplinervium* C. DC.	MAJ 139	AF275168	DUKE	Jaramillo & Manos (2001)
***Pipernokaidoyitau***	**WT 4171**	**OK235339**	**COAH**	**This study**
	**WT 4258**	**OK235340**	**COAH**	
	**ET 7249**	**OK235341**	**COAH**	
*Pipernovogranatense* C. DC.	MAJ 71	EU581317	DUKE	Jaramillo et al. (2008)
*Piperpeltatum* L.	MAJ 564	EU581335	DUKE	Jaramillo et al. (2008)
*Piperretrofractum* Vahl.	MAJ 395	AF275196	DUKE	Jaramillo & Manos (2001)
*Pipersanctum* (Miq.) Schltdl. ex C. DC.	AB 744	EU581382	SEMO	Jaramillo et al. (2008)
*Piperscutilimbum* C. DC.	WT 4151	OK235343	UMNG	This study
*Piperscutifolium* Yunck.	MAJK 281	EU581389	SPF	Jaramillo et al. (2008)
*Pipersorgosonum* C. DC.	MAJ185	AY572320	DUKE	Jaramillo & Manos (2001)
*Piperspoliatum* Trel. & Yunck.	MAJ 60	AF275179	DUKE	Jaramillo & Manos (2001)
*Pipersubscutatum* C. DC.	EJT 1604	EU581406	MU	Jaramillo et al. (2008)
*Pipertrianae* C. DC.	MAJ 662	EU581413	DUKE	Jaramillo et al. (2008)
*Pipertruncatum* Vell.	MAJ 937	EF056291	RB	Jaramillo et al. (2008)
*Pipertuberculatum* Jacq.	MAJ 710	AY326225	DUKE	Jaramillo & Callejas (2004)
*Piperumbellatum* L.	MAJ 35	EU581433	DUKE	Jaramillo et al. (2008)
*Piperumbricola* C. DC.	EJT 1014	EU581435	MU	Jaramillo et al. (2008)
***Pipervelae***	**WT 4058**	**OK235344**	**UMNG**	**This study**
	**WT 3995**	**OK235345**	**UMNG**	
*Piper viçosanum* Yunck.	MAJ 809	EU581440	RB	Jaramillo et al. (2008)
*Piperyanaconasense* Trel. & Yunck.	MAJ 774	AY326229	DUKE	Jaramillo & Callejas (2004)

*Collections: COAHH, Herbario Amazónico Colombiano; DUKE, Duke University Herbarium, UMNG: Herbario Universidad Militar Nueva Granada; HUA, Herbario de la Unviersidad de Antioquia; INPA, Instituto Nacional de Pesquisas da Amazônia; MG, Museu Paraense Emílio Goeldi; MU, Miami University Herbarium; NY, New York Botanical Garden Herbarium; RB, Herbario Jardim Botânico do Rio de Janeiro; SEMO, Southeast Missouri State University Herbarium; SPF, Herbario da Universidade de São Paulo; SRP, Snake River Plains Herbarium (University of Idaho).

## ﻿Results

**Figure 1.**
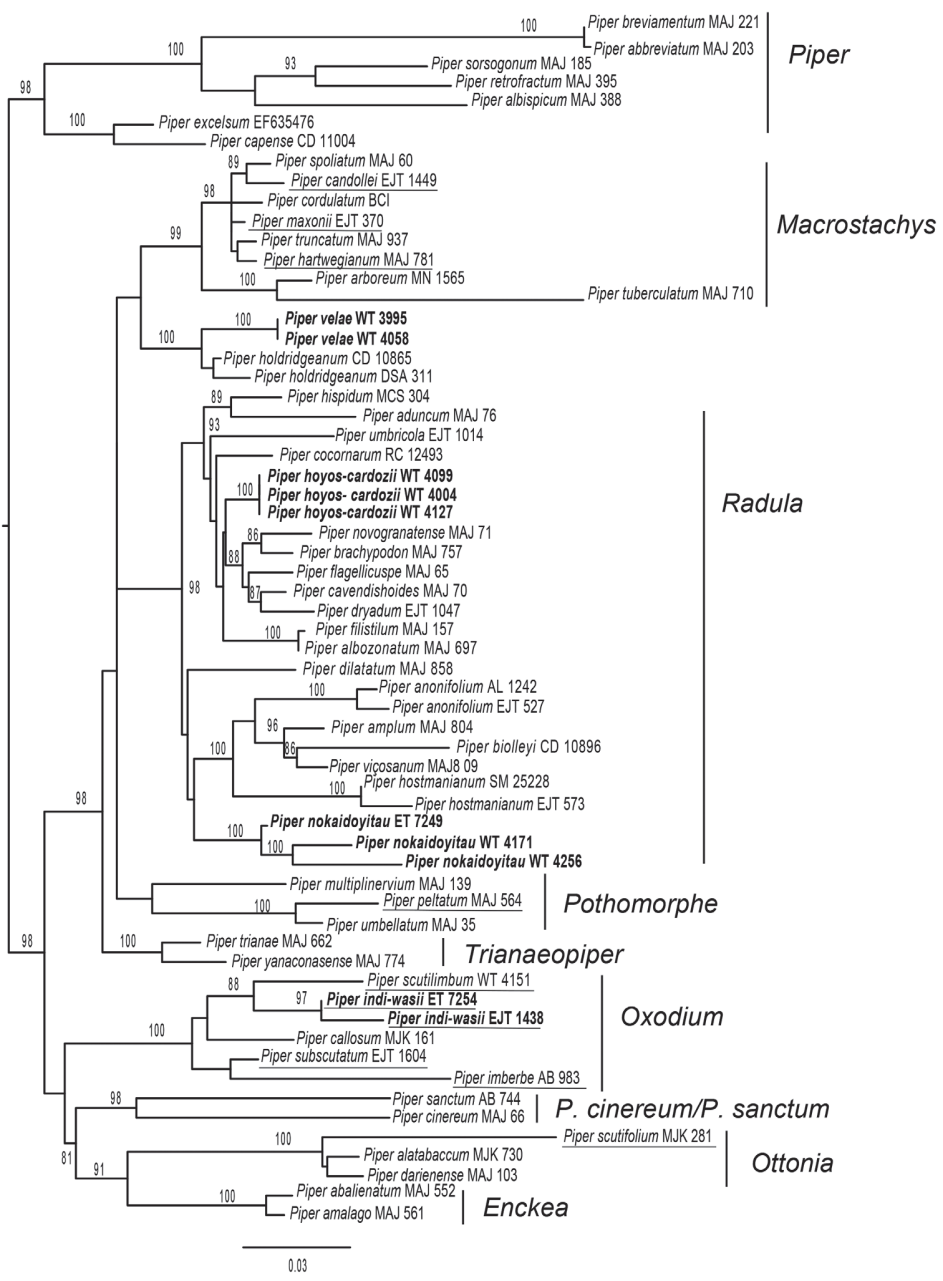
Phylogenetic relationships of species described in this manuscript, based on Maximum Likelihood analysis of nrITS sequences. New species are shown in bold. Species with peltate leaves are underlined. Numbers above branches are ML bootstrap support values (> 80).

Phylogenetic analyses identified the relationships of the new species described here (GenBank accession numbers are provided in Table 1). The ML tree placed *Piperhoyoscardozii* and *Pipernokaidoyitau* within the *Radula* clade, *Piperindiwasii* species within *Oxodium* (= *Schilleria*, Callejas-Posada 2020) and *Pipervelae* forms a clade with *Piperholdridgeanum* W. C. Burger (1971: 144–145), sister to the *Macrostachys* clade (Fig. 1).

### ﻿Taxonomic treatment

#### ﻿ Piper hoyoscardozii

Taxon classificationPlantaePiperalesPiperaceae

W. Trujillo-C & M. A. Jaram. sp. nov.

92B8727A-6424-595F-B81F-BE14E19F1C47

urn:lsid:ipni.org:names:77303983-1

**Figure 2.**
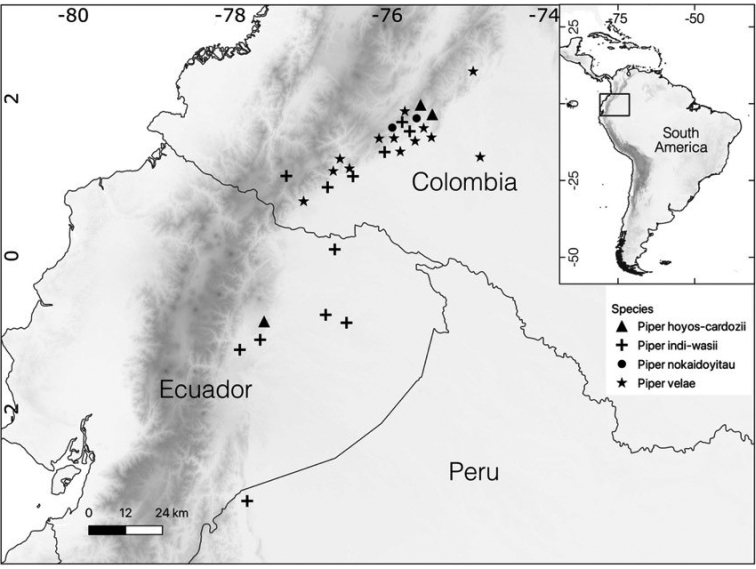
The locality of four new *Piper* species from the Andes eastern slopes of Colombia, Ecuador and Peru.

**Figure 3.**
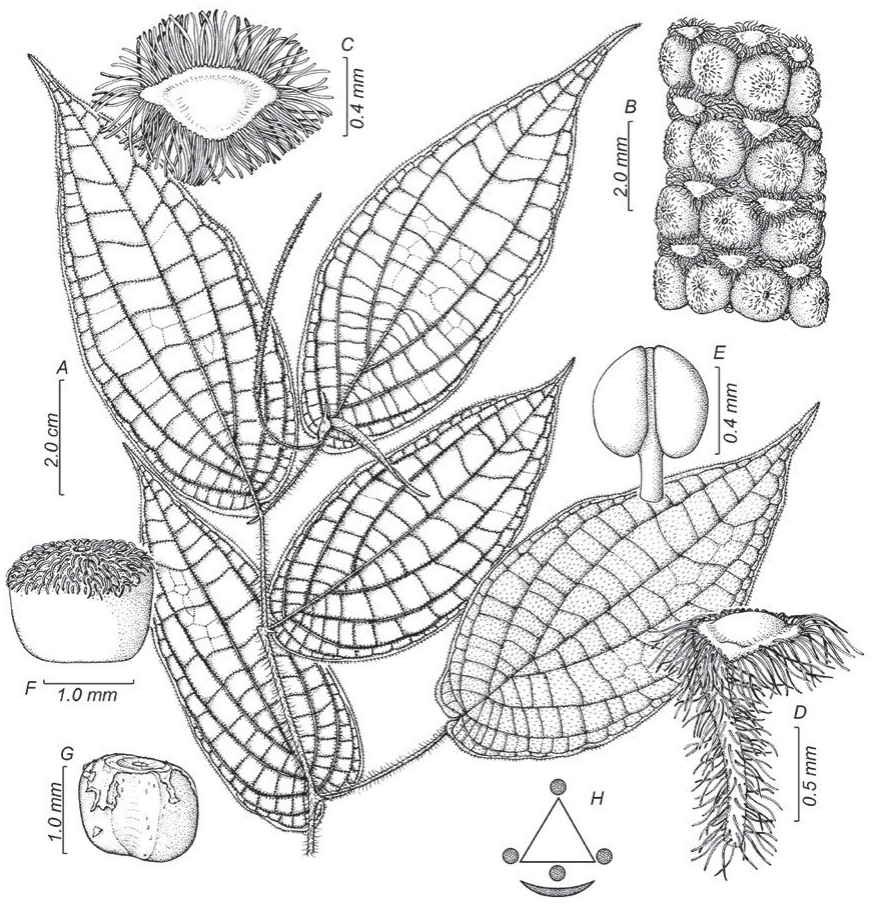
*Piperhoyoscardozii***A** sympodial branch **B** magnified view of the infrutescence **C** floral bract view from above **D** floral bract side view **E** anthers and distal portion of the filament **F** fruit side view **G** seed **H** floral diagram. Illustration by Marcela Morales based on *F. Hoyos 049* COAH.

**Figure 4.**
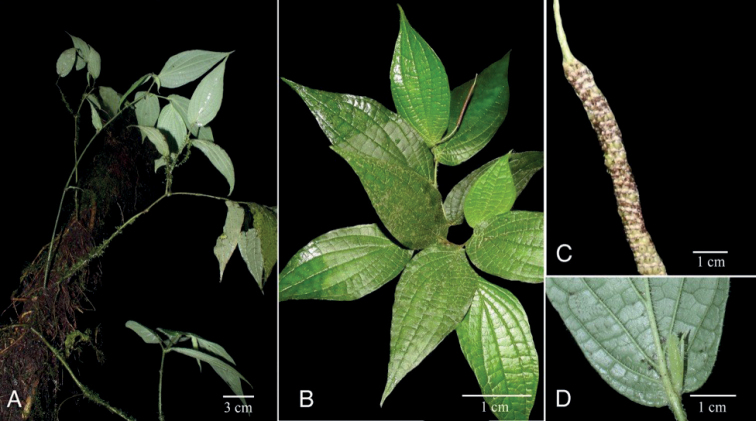
*Piperhoyoscardozii***A** growth form, scandent shrub **B** upper leaf surfaces and spike **C** infructescence **D** leaf base and prophyll. Photos from *F. Hoyos 049* by F. Hoyos

Figs 1 , 2 , 3 and 4

##### Type.

Colombia. Caquetá: Florencia, vereda Sucre, 1076 m elev., 1°47'50"N, 75°38'50"W, 18 Oct 2020 [fr], *F. Hoyos 049* (Holotype COL, Isotype COAH, HUA, UMNG)

##### Diagnosis.

*Piperhoyoscardozii* W. Trujillo & M. A. Jaram. is similar to *P.dryadum* C. DC. (1891:221) and *P.flagellicuspe* Trel. & Yunck. (1950:59) from which it is easily distinguished by peduncles 2–3 cm long, spikes long-apiculate and fruit with stigmas sessile vs. peduncle 0.5–1 cm long, spikes not-apiculate and fruit with stigmas on a short style in *P.flagellicuspe* and *P.dryadum*.

##### Description.

***Shrub*** with sarmentose branches. Internodes (1)3–7 cm long, smooth, green, pubescent, trichomes pluricellular, uniseriate, 1–2.3 mm long, idioblasts not visible. ***Prophylls*** caducous, 1.2–1.5 cm long, green-whitish, pubescent, trichomes pluricellular, uniseriate, 0.2–1.0 mm long, dispersed on the abaxial surface, idioblasts not visible. ***Petioles*** uniform in size along all axes, 0.5–0.8 cm long, vaginate on the basal half, smooth and pubescent. ***Leaf-blades*** membranaceous, drying black, uniform in shape and size on all nodes, (5)6–7 × (13)15–17 cm, elliptic, symmetric, base cordate to rounded, apex acuminate; leaf blade smooth, pubescent on both surfaces, trichomes pluricellular, uniseriate, 0.5–2.3 mm long, dispersed on the adaxial surface, along first and second order nerves and dispersed on the areolas and third order nerves of the abaxial surface, eciliate; pinnately nerved from the lower 1/3, 2–3 nerves on each side, with spacing uniform or decreasing and angle increasing gradually towards the base, eucamptodromous, tertiary veins percurrent. ***Inflorescence*** and ***infructescence*** a simple spike, erect; peduncle 2–3 cm long, pubescent, green; rachis in flower 4–7 cm long, rachis in fruit 7–9 cm long, rachis with a 10–15 mm long, sterile green apical extension, fruits densely grouped along the rachis. ***Floral bracts*** cucullate, reddish in flower, triangular from above, 0.4–0.6 × 0.7–0.8 mm, glabrous on the adaxial surface, margin fimbriate, bracts forming bands around the spike. ***Flowers*** with four stamens, filaments 0.6–0.8 mm long, anthers 0.4–0.6 × 0.5–0.7 mm, longitudinally dehiscent, dithecous, with connective not protruding, glabrate, idioblasts not evident, black when dried. Sessile stigmas. ***Fruits*** rectangular, laterally compressed, green when alive and black when dry, 0.9–1.2 × 1.5–1.9 mm, pubescent, partially immersed in the rachis, with persistent sessile stigmas, 0.05–0.1 mm. Seeds oblong, laterally compressed, brown, smooth, 0.8–1 × 0.8–1.1 mm.

##### Distribution and habitat.

*Piperhoyoscardozii* is a shade-loving sarmentose shrub that grows on trees and rocks. It is known from the Amazonian slope of the Andes in southern Colombia and Ecuador, between 1000–1500 m in elevation (Fig. 2).

##### Phenology.

Flowering specimens were collected in July. Fruiting specimens were collected in August.

##### Etymology.

This species name is dedicated to Fernando Hoyos Cardozo, a great companion during our floristic explorations of the Amazonian foothills and who collected the type specimen of this species in Caquetá.

##### Conservation status.

This species is known from six specimen collections representing two subpopulations. The locations where it occurs are threatened by deforestation and expansion of the agriculture frontier, especially extensive cattle ranching. The extent of occurrence (EOO) of 876 km^2^ and area of occupancy (AOO) of 16 km^2^ are small, which, together with the continuing decline in quality of habitat, suggests it is Endangered [EN B1a+B2a].

##### Phylogenetic relationships.

*Piperhoyoscardozii* belongs to the large clade *Radula*. A group of medium-size shrubs, mostly self-supporting, but some species are herbs or lianescent shrubs; leaves are plinerved or pinnately nerved. Flowers are densely arranged in spikes forming banding patterns and inflorescences can be erect or distally curved. Furthermore, this species is closely related to the clade of sarmentose shrubs occurring in wet tropical forests in Central America and the western slopes of the west Cordillera in Colombia (the latter corresponds largely to the Chocó Region) that includes *P.brachypodon* C. DC. (1869:327), *P.cavendishioides* Trel. & Yunck. (Trelease and Yuncker 1950: 85), *P.dryadum*, *P.flagellicuspe*, *P.ottoniifolium* C. DC. (1866:213), *P.oxystachyum* C. DC. (1898:255) and *P.novogranatense* C. DC. (1869:313) (Jaramillo et al. 2008).

##### Discussion.

*Piperhoyoscardozii* is a sarmentose shrub, a habit not commonly observed amongst *Piper* species in the study region (eastern slope of the Andes). The phylogeny (Fig. 1) places *P.hoyoscardozii* sister to other climbing *Piper* species occurring on the western slope of the Andes and wetter parts of Mesoamerica. Here we provide a comparative table for the climbing *Piper* species included in the phylogeny (Table 2). *P.hoyoscardozii* is easily differentiated because its spikes (in flower and fruit) have a long peduncle and a long apiculate apex.

**Table 2.** Comparison of *Piperhoyoscardozii* with related species of scandent habit.

Species	Internodes indument	Secondary nerves branch	Peduncle length	Rachis length	Spike a pex	Distribution
***P.brachypodon* (Benth.) C. DC.**	glabrous	from the lower half	1 cm	5–7 cm	obtuse	Chocó Region
***P.cavendishioides* Trel. & Yunck.**	tomentulose	from the lower one-fourth or one-third	1 cm	7–8 cm	obtuse	Chocó Region
***P.dryadum* C. DC.**	pubescent	from the lower third	0.5–1 cm	5–6 cm	obtuse	Mesoamerica and Chocó Region
***P.flagellicuspe* Trel. & Yunck.**	velvety	from the lower half	0.5 cm	4 cm	obtuse	Chocó Region
***P.novogranatense* C. DC.**	glabrous	from the lower third	0.5 cm	4 cm	obtuse	Chocó Region
***P.ottoniaefolium* C. DC.**	glabrous	near the base	0.5–1 cm	6–8 cm	obtuse	Chocó Region
***P.xanthostachyum* C. DC.**	glabrous	from the lower third	0.5–1 cm	5–6.5	obtuse	Mesoamerica and western slope of the Andes
***P.hoyoscardozii* W. Trujillo & M. A. Jaram.**	pubescent	from the lower third	2–3 cm	7–9 cm	long apiculate	Eastern slope of the northern Andes

##### Specimens examined.

**Colombia**: Caquetá, Florencia, vereda Tarqui, monumento Divino Niño, 1570 m elev., 1°50'0.3"N, 75°39'52.8"W, 30 Aug 2020 [fl], *W. Trujillo & F. Hoyos 4120* (COAH, UMNG).; vereda Tarqui, quebrada Tarqui, 1530 m elev., 1°50'28"N, 75°39'42"W, 20 Aug 2020 [42], *W. Trujillo 4099* (COAH); corregimiento El Caraño, vereda Sucre. 1076 m elev., 1°47'50.8"N, 75°38'50.5"W, 8 Jul 2014 [fl], *W. Trujillo 3130* (COAH); vereda Sucre, Finca campamento Sucre. 1°46'52"N, 75°39'5.1"W. 1050 m elev,. 5 Jul 2012 [fl]. *W. Trujillo & C. Malambo 2400*; vereda Sucre, vía antigua Florencia-Huila, 1°47'50.8"N, 75°38'50.3"W, 1000 m elev., 24 Sep 2020 [fl], *F. Hoyos 042* (COAH). **Ecuador.** Napo, Parque Nacional Sumaco-Galeras, 0°50'S, 77°34'W, 1090 m elev., 27 Oct 2005 [fr], *J. Homeier & M.A. Chinchero 2000* (MO).

#### ﻿ Piper indiwasii

Taxon classificationPlantaePiperalesPiperaceae

W. Trujillo-C & M. A. Jaram. sp. nov.

138BB73E-0D57-5BDD-893D-64DB4B7025B4

urn:lsid:ipni.org:names:77303984-1

**Figure 5.**
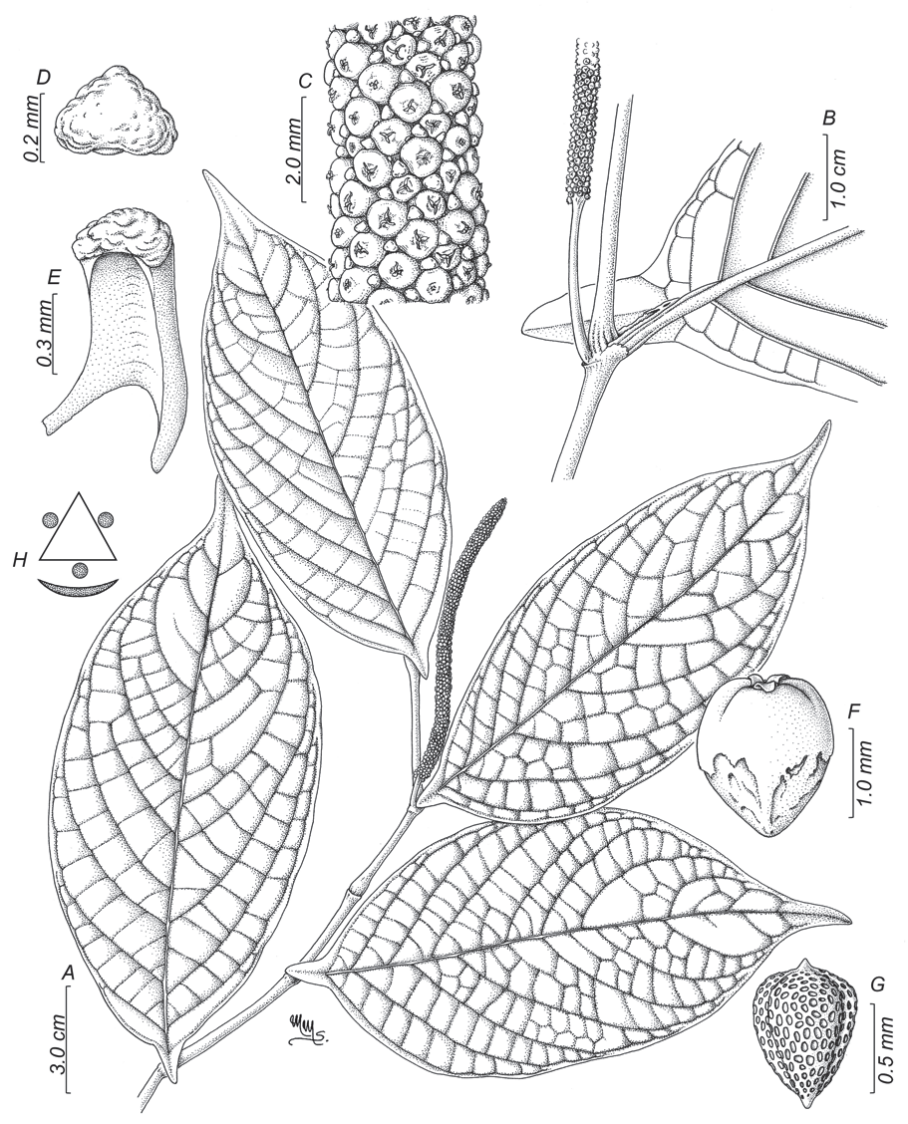
*Piperindiwasii***A** sympodial branch **B** lower leaf surfaces and details of the petiole **C** magnified view of the infructescence **D** floral bract view from above **E** floral bract side view **F** fruit side view **G** seed side view. Illustration by Marcela Morales based on *Angulo 1550*, COAH.

**Figure 6.**
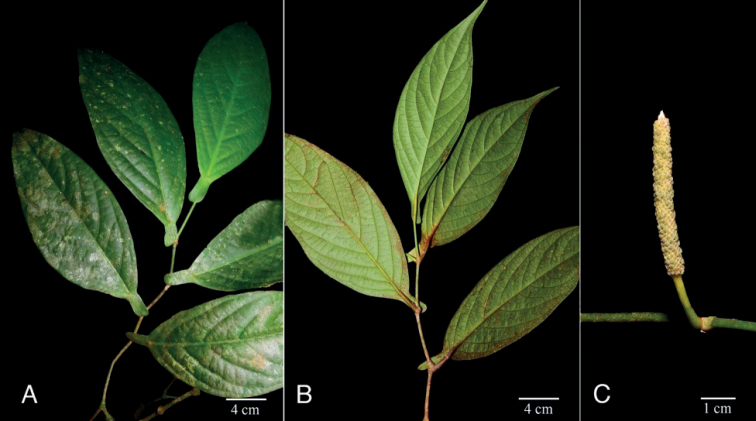
*Piperindiwasii***A** sympodial branch upper leaf surfaces **B** sympodial branch lower leaf surfaces **C** erect spikes. Photos from *E. Trujillo 7249* by G. Delgado.

Figs 1 , 2 , 5 and 6

##### Type.

Colombia. Caquetá, municipio de San José del Fragua, ronda de bosque cerca al balneario Villa Collazos sobre el rio Fragua, 1°20'04"N, 75°59'28"W, 395 m elev., 14 May 2020, *M. Angulo 1550* (Holotype COL, Isoptype COAH, HUA, HUAZ, UMNG).

##### Diagnosis.

*Piperindiwasii* W. Trujillo & M. A. Jaram. can be distinguished from *P.scutilimbum* C. DC. (1920a:242) by many attributes. *Piperindiwasii* has 1–1.7 cm long petioles, 7–8 pairs of secondary veins and a narrowly spatulate leaf base extension, 0.4–0.9(1.5) cm wide, vs. *P.scutilimbum*, which has a 4–6 cm long petioles, 10–12 secondary veins and an obtuse and rounded leaf-base extension, 2.5–4 cm wide. *Piperindiwasii* occurs in the Amazon watershed, on the eastern foothills of the Andes, while *P.scutilimbum* occurs west of the Andes in Panama and extends to Sierra Nevada de Santa Marta in northern Colombia.

##### Description.

***Shrub***, up to 2 m tall. Internodes 2–4(5) cm, canaliculate, green, glabrous. ***Prophylls*** not seen. ***Petioles*** are uniform in size along all axes, 1–1.7 cm long, vaginate along their entire length, canaliculate, glabrous. ***Leaf-blades*** coriaceous, drying grey to brown, uniform in size along all axes, (4.5)6–8.5 × (11)14–18 cm, elliptic, symmetric, leaf-base peltate, symmetric, with a narrowly spatulate extension, 0.4–0.9 × (0.5)1–2.3(2.5) cm, the leaf-base extension covering the petiole on sympodial nodes and orientated towards the axis on monopodial nodes, apex acuminate; leaf-blade glabrous on both surfaces, eciliate; pinnately nerved throughout, the nerves 7–8 on each side, brochidodromous, with spacing decreasing towards the base and angle uniform throughout, tertiary veins random reticulate. ***Inflorescences and infructescences*** a solitary spike, erect; peduncle 0.9–1.5 cm long, glabrous, green; rachis length in fruit (5)6–20 cm, fruits loosely grouped along the rachis. ***Floral bracts*** cucullate, triangular from above, 0.3–0.5 × 0.5–0.8 mm, glabrous on the adaxial surface, margin eciliate, not forming bands around the spike. ***Flowers*** with three stamens, filaments 0.5–0.8 mm long, anthers 0.3–0.5 × 0.2–0.3 mm long, longitudinally dehiscent, dithecous, with connective not protruding, glabrate, idioblasts not evident; stigmas 3, 0.05–1.5 mm long, sessile. ***Fruits*** obpyriform, green when alive and brown when dry, 0.6–0.8 × 0.9–1.2 mm, glabrous, partially immersed in the rachis, with stigmas persistent, 0.05–1.5 mm long, sessile. ***Seeds*** obpyriform, black.

##### Distribution and habitat.

*Piperindiwasii* is known from the Amazonian slopes of the Andes in Colombia (Departments of Putumayo, Caquetá and Guaviare), Ecuador (Provinces of Guayas, Napo, Orellana, Pastaza and Sucumbíos) and Peru (Provinces of Amazonas), from 200 to 1,608 m elevation (Fig. 2). It occurs in lowland (sometimes along riverbanks) and lower montane forests. It is a shade-loving species that grows in the understorey and the edges of trails of preserved forests.

##### Phenology.

Fruiting specimens were collected from December to June and August to October. Flowering samples were collected in March, April, May and December.

##### Etymology.

*Piperindiwasii* is named after the Inga word meaning “House of the Sun”. Ingas or Inganos are an indigenous group belonging to the Quechua linguistic family. The Ingas of the Amazon foothills are made up of migratory groups from the Peruvian and Ecuadorian Amazon, the Mocoas and some survivors of the Andaquíes. The clans are united by their location, cosmovision and the culture of “yajé” (*Banisteriopsiscaapi*) . Furthermore, the type specimen of this species was collected in the Alto Fragua Indi-Wasi National Park, located in San José del Fragua, Caquetá-Colombia.

##### Conservation status:

*Piperindiwasii* is not endangered. It is known from 11 subpopulations and 12 localities; it has an EOO of 1,478,359 km^2^ and an AOO of 52 km^2^. According to IUCN guidelines, it is of Least Concern (LC) as the region where it occurs is threatened by deforestation and its conservation status should be monitored.

##### Phylogenetic relationships.

*Piperindiwasii* belongs to the Neotropical clade *Oxodium*. Species in this group are shrubs, sometimes sarmentose. They have plinerved or pinnately nerved leaves, leaf bases are often acute or cordate and they have lax inflorescences with loosely arranged flowers (Jaramillo et al. 2008). *Piperindiwasii* is closely related to *P.scutilimbum*. Nucleotide difference between the two *Piperindiwasii*ITS accession is 5 bp out of 641 (0.8%), while these sequences have a 27 bp (4%) dissimilarity with *P.scutilimbum* (collected in the type locality). Sequence data and leaf material were available for five species of Neotropical *Piper* with peltate leaves. *P.subscutatum* C. DC. (1869:321) and *P.imberbe* Trel. & Standl. (Standley and Steyermark 1952:303–304) are in the *Oxodium* clade as *P.indiwasii* and *P.scutilimbum*; *P.scutifolium* Yunck. (1966:123–124) is part of the *Ottonia* clade; and *P.maxonii* C. DC. (1920b:16), *P.hartwegianum* (Benth.) C. DC. (1869: 369) and *P.candollei*Sodiro (1905:202) are members of the *Macrostachys* clade. Neotropical *Piper* species with peltate leaves do not form a monophyletic group and are part of at least four clades: *Oxodium*, *Pothomorphe*, *Macrostachys* and *Ottonia* (Fig. 1). Further studies are needed to shed light on the convergence of this trait.

##### Uses by communities.

Various common names are used for *P.indiwasii* amongst indigenous communities in Ecuador: a) “*ñahui tapa panga*” (closed eye leaf) (D. Irvine & L. Cejua 1125, F, QCA); when a patient is sick and the eyes are closed even when awake, leaves are wrapped around tobacco and the smoke blown over the eyes of the patient; b) “uchi-ampar” a Shuar name, plant used against parasites, leaves and roots are used (Guerrero 171; Herrera 288, MO, QCNE); c) “*palu sera aula*” or the grandmother of *palu sera*, which is used for relieving toothache (D. Irvine 959, F, QCA).

##### Discussion.

After reviewing the specimens identified as *P.scutilimbum* from Panama and northern Colombia vs. specimens from the eastern (Amazonian) Andes slope in Colombia, Ecuador and Perú (Fig. 2), we found a consistent difference in the leaf base shape between collections from both regions. The obtuse and rounded leaf base extension of the type specimen from the Sierra Nevada de Santa Marta coincides with that of specimens collected in Panama in contrast to the narrowly spatulate base extension seen in *P.indiwasi*. The leaf base shape, combined with other morphological characters and geographical distribution, clearly allows *Piperindiwasii* to be proposed as a new species. We provide a comparative table of morphological characters for species of *Piper* with peltate leaves that belong to the *Oxodium* clade (see Table 3).

**Table 3.** Comparison of *Piperindiwasii* with species of *Piper* with peltate leaves belonging to the *Oxodium* clade.

Species	Leaf dimorphism*	Leaf base extension width	Leaf base extension shape	Leaf width	Geographic distribution
***P.imberbe* Trel. & Standley**	all leaves peltate	1.2–1.5 cm	rounded	4.5–8 cm	Mesoamerica
***P.scutilimbum* C.DC.**	all leaves peltate	(2)2.5–4 cm	obtuse and rounded	8–14 cm	Mesoamerica and northern Andes
***P.subscutatum* (Miq.) C. DC**	present	2–4 cm	obtuse and rounded	25–28 cm	Eastern slope of the northern Andes and Amazonia
***P.indiwasii* W. Trujillo & M. A. Jaram.**	all leaves peltate	0.4–0.9 cm	narrowly spatulate	4–18 cm	Eastern slope of the northern Andes

* Leaves can be peltate or not.

##### Specimens examined.

**Colombia**. Caquetá, San José del Fragua, ronda de bosque cerca al balneario Villa Collazos sobre el rio Fragua, 1°20'04"N, 75°59'28"W, 400 m elev., 29 Jun 2011 [fr], *W. Trujillo et al. 1999* (COAH); Belén de los Andaquies, vereda las verdes, cerro Monserrate, entrada por dos quebradas, 1°36'38"N, 75°53'23"W, 700 m elev., 24 Jun 2011 [fr], *W. Trujillo et al. 1990* (COAH); Belén de los Andaquies, Parque Natural Municipal Andaqui, cabeceras del rio pescado, 1°41'52"N, 75°54'15"W, 1608 m elev., 25 Jan 2017 [fr], *N. Castaño et al. 8734* (COAH, HUA); Belén de los Andaquies, Parque Natural Municipal Andaqui, sector entre filo seco y la bocana de la quebrada las verdes, 1°37'13"N, 75°53'46"W, 600–800 m elev., 7 Feb 2017 [fr], *N. Castaño et al. 9659* (COAH, HUA); Florencia, vereda Damas Arriba, finca el Mirador, 1°37'56"N, 75°41'49"W, 750 m elev., 14 Feb 2002 [fr], *M. Correa et al. 2853* (COAH, UDBC); Guaviare, El Retorno, cerca del Retorno, granja de la Corporación Araracuara, zona ligeramente disectada, bosque intervenido, 1 Mar 1994 [fl], *P. Stevenson 1168* (COAH); Putumayo, Villagarzón, corregimiento la Castellana, vereda la Pradera, finca el Cairo, bosque intervenido a borde de quebrada, 0°52'23"N, 76°45'27"W, 600 m elev., 10 Dec 1999 [fr], *C. Marín & D. Cárdenas 1997* (COAH); Villagarzón, vereda la Kofaina, 1°01'00"N, 77°17'00"W, 550–700 m elev., 2 Sep 1993 [fr], *A. Cogollo et al. 6830* (COAH, JAUM, MO); **Ecuador**. Napo, Estación Biológica Jatun Sacha, 1°04'00"S, 77°37'00"W, 450 m elev., 10 Oct 2007 [fr], *J. Homeier et al. 2834* (MO, QCA, QCNE, GOET); Estación Biológica Jatun Sacha, rio Napo, 8 km debajo de Misahualli, 1°04'00"S, 77°36'00"W, 450 m elev., 17 Jan – 6 Feb 1987 [fr], *C. Cerón 638* (MO, HUA); Estación Biológica Jatun Sacha, rio Napo, 8 km debajo de Misahualli, 1°04'00"S, 77°36'00"W, 450 m elev., 19–28 Mar 1987 [fr], *C. Cerón 1063* (HUA, MO); 9 km rio debajo de puerto Misahualli y 2 km al sur de la cuenca del rio Chinguipino, 1°05'00"S, 77°36'00"W, 430 m elev., 10 Mar 1985 [fr], *D. Neill et al. 6054* (ECUAMZ, HUA, MO, NY, QCNE, US); Tena, Estación Biológica Jatun Sacha, rio Napo, 8 km E of puerto Misahualli, 1°04'00"S, 77°36'00"W, 400 m elev., 18 May 1985 [fr], *W. Palacios 421* (AAU, HUA, MO, NY, QCNE, US); Tena, Estación Biológica Jatun Sacha, along S bank of rio Napo, 8 km E of puerto Misahualli, 1°04'00"S, 77°36'00"W, 450 m elev., 1 Apr 1992, *T. Croat 73366* (HUA, MO) [fr]; along road between Tena and Puyo, 61.5 km N of Puyo, 1°11'36"S, 77°52'34"W, 500 m elev., 22 Dec 1979 [fl], *T. Croat 49657* (MO); Archidona, Parque Nacional Galeras a 1.5 km de la comunidad Santa Rosa de Arapino, 00°51'00"S, 77°31'00"W, 1230 m elev., 3 Apr 1996 [fr], *H. Vargas & P. Grefa 951* (HUA, MO, QCNE); San José de Payamino 40 km W of Coca, 00°30'S, 77°20'W, 26 Apr 1984 [fl], D. Irvine & H. Jipa 959 (F, QCA); 1 May 1984, D. Irvine & L. Cejua 1125 (F, MO, QCA); Sucumbíos, Dureno, comunidad Cofan al sur del rio Aguarico, 20 km al este de Lago Agrio, 00°05'00"N, 76°40'00"W, 350 m elev., 27 Dec 1988 [fr], *C. Cerón et al. 5824* (MO, HUA); Dureno, comunidad Cofan al sur del rio Aguarico, 20 km al este de Lago Agrio, 00°05'00"N, 76°40'00"W, 350 m elev., 27 Dec 1988 [fr], *C. Cerón et al. 5827* (MO, HUA); Francisco de Orellana, Orellana, comunidad Shuar Tiguano al sur del Coca por la vía al Pindo, 00°44'58"S, 76°46'55"W, 300 m elev., 6–12 May 2004 [fl], *W. Guerrero & A. Herrera 171* (MO, QCNE); Orellana, comunidad Shuar Tiguano al sur del Coca por la vía al Pindo, 00°44'58"S, 76°46'55"W, 300 m elev., 11 May 2004 [fr], *A. Herrera & W. Guerrero 288* (MO, QCNE); Parque Nacional Yasuni, Rio Tiputini al noroeste de la confluencia con el Rio Tivacuno, este de la carretera Repsol-YPF, km 32 hacia NPF, Sendero Botánico Guiyero, 00°38'S, 76°30'W, 200—300 m elev., 26 Feb 2002 [fr], *G. Villa 1350* (F, QCA, US); **Peru**. Amazonas, Condorcanqui, Santiago, Cerros Kampankis, Serranía entre los Rios Santiago y Morona, desde Río Marañón hasta frontera con Ecuador, Campamento 1: Pongo Shenin, 03°07'01.52"S, 77°46'55.14"W, 520 m elev., 3 Aug 2011 [fr], *I. Huamantupa 15217* (USM, F).

#### ﻿ Piper nokaidoyitau

Taxon classificationPlantaePiperalesPiperaceae

W. Trujillo-C & M. A. Jaram. sp. nov.

6726D4AE-2BBF-5514-9CAE-A0CC4C3EAF5D

urn:lsid:ipni.org:names:77303985-1

**Figure 7.**
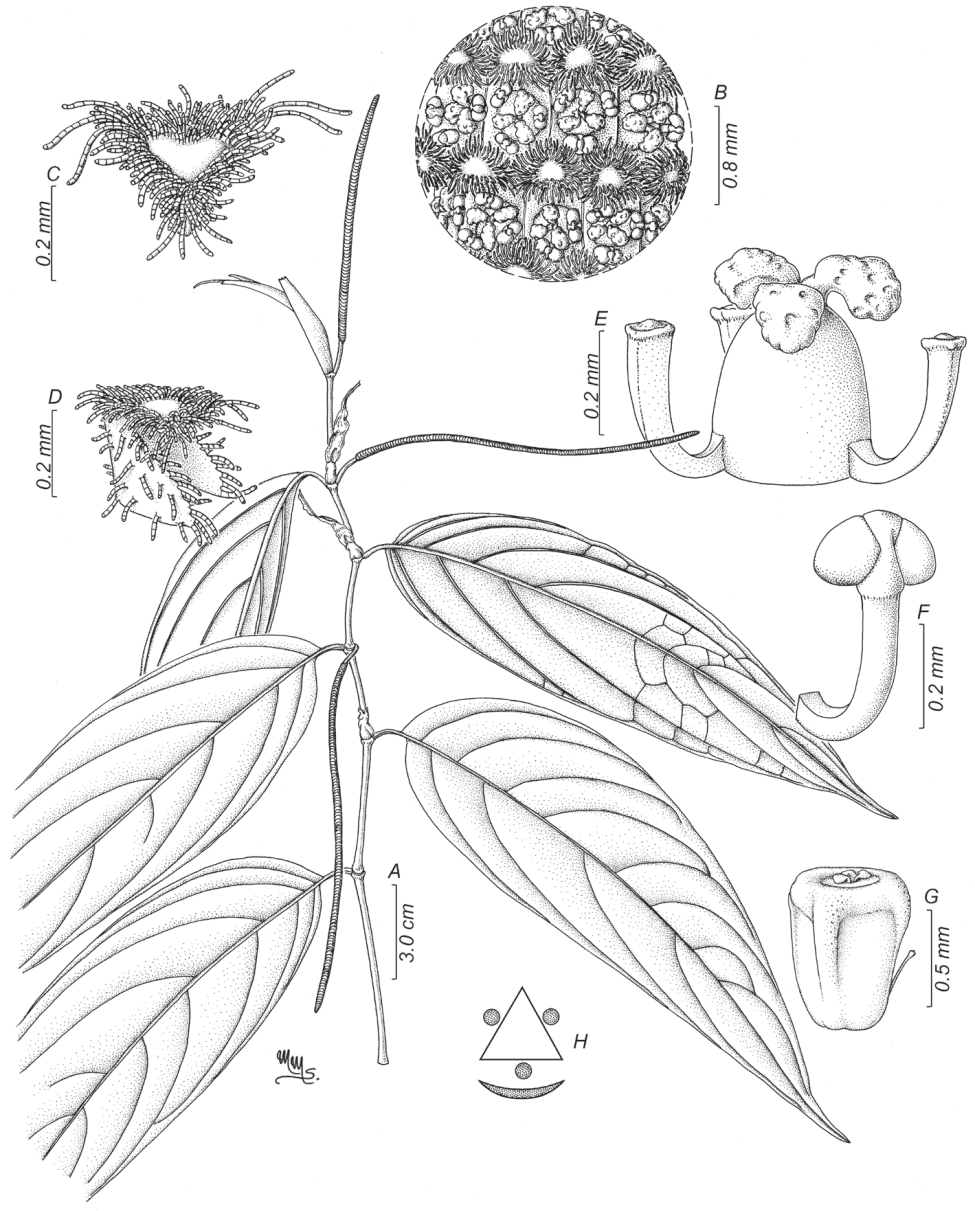
*Pipernokaidoyitau***A** sympodial branch **B** details of the inflorescence **C** floral bract viewed from above **D** floral bract side view **E** flower side view **F** stamen with glabrous connective **G** fruit side view **H** floral diagram. Illustration by Marcela Morales based on *F. Hoyos 046* (COL).

**Figure 8.**
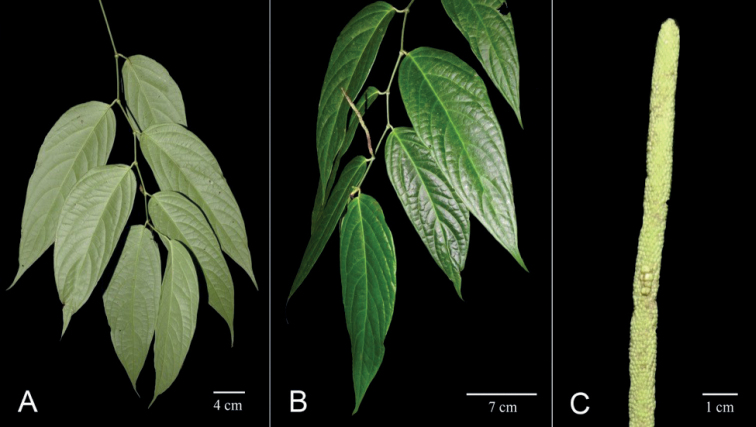
*Pipernokaidoyitau***A** sympodial branch, lower leaf surfaces **B** sympodial branch, upper leaf surfaces and spikes **C** single infructescence. Photos from *F Hoyos & W Trujillo 046* by F Hoyos

Figs 1 , 2 , 7 and 8

##### Type.

Colombia, Caqueta; Florencia, corregimiento el Caraño, vereda Sucre, 01°47'50.8"N, 75°38'50.5"W, 1020 m elev., 25 Oct 2020 [fr], *F. Hoyos & W. Trujillo 046* (Holotype COL, Isotype COAH, UMNG, HUAZ, HUA)

##### Diagnosis.

*Pipernokaidoyitau* W. Trujillo & M. A. Jaram., can be separated from the similar species *P.hostmannianum* (Miq.) C. DC. (1869:287), by its prophylls up to 2.4 cm long, leaves 12–20 cm long vs. prophylls 2.8–3.5 cm long, leaves 21–26 cm long in *P.hostmannianum*.

##### Description.

***Shrub*** up to 3 m tall; internodes (2.5–)3–4.5 cm long, canaliculate superficially, green, glabrous. ***Prophylls*** 2.8–3.2 cm long, green, glabrous, caducous, swollen in the basal portion (observable in live plants). ***Petioles*** uniform in size along all nodes, (0.7–)1–1.2(–1.5) cm long, sheathing at the base, smooth, glabrous. ***Leaf-blades*** coriaceous, drying black, uniform in shape and size along all axes, (4.5–)5–8 × (18) 21–26 cm, ovate, asymmetric, base rounded, glabrous on both surfaces, eciliate; pinnately nerved throughout, 4–5 ascending nerves on each side, eucamptodromous, with spacing decreasing and angle increasing towards the base, tertiary veins random, reticulate; apex acuminate. ***Inflorescences*** simple spikes, erect; peduncle 1–1.5 cm long, glabrous, green; rachis (7.5)10–12 × 0.3 cm in flower, 11–13 × 0.4–0.5 cm in fruit, flowers densely grouped along the rachis, forming bands around the spike. Floral bracts cucullate, heart-shaped from above, 0.2–0.35 × 0.4–0.7 mm, glabrous centrally on the abaxial surface, margin densely fimbriate. ***Flowers*** with three stamens, filaments 0.3–0.5 mm long, anthers 0.1–0.25 × 0.2–0.3 mm, transversally dehiscent, dithecous, with connective not protruding, glabrate, idioblasts not evident, colour black when dried; stigmas 3, 0.1–0.25 mm long, sessile. ***Fruits*** obpyriform in side view and triangular from above, green when alive, black when dry, 0.5–0.7 × 0.8–0.9 mm, glabrous, partially immersed in the rachis, with stigmas sessile and persistent.

##### Distribution and habitat.

*Pipernokaidoyitau* is known from the lower montane forests in the eastern slopes of the Andes in Colombia, ca. 1,100 m elevation (Fig. 2), the Department of Caquetá. It is a shade-loving species that grows in the understorey of preserved forest.

##### Phenology.

Flowering specimens were collected in September and October. Fruiting specimens in October.

##### Etymology.

*Pipernokaidoyitau* is named after the Huitoto name for *Piper* plants, “Nokaido yitau”. It means “the powers of the toucan” because these are sacred and medicinal plants used against fever, body and headaches and as anti-inflammatories.

##### Phylogenetic relationships.

*Pipernokaidoyitau* belongs to the *Radula* clade of Neotropical *Piper* (Jaramillo et al. 2008). Specifically, *P.nokaidoyitau* is sister to the *Isophyllon* subclade, within *Radula*. *Isophyllon* species are mostly self-standing shrubs with coriaceous leaves, pinnately nerved, acute or obtuse bases and flowers densely arranged in erect inflorescences. *Isophyllon* species occur in the Atlantic Forest, Central America and the Amazon Region.

##### Conservation status.

This species is known from four specimen collections representing two subpopulations. The locations where it occurs are threatened by deforestation and expansion of the agriculture frontier. The area of occupancy (AOO) of 8 km^2^ is small, which, together with the continuing decline in habitat quality, suggests it is Endangered [EN B1a+B2a].

##### Comments.

Leaves of *Pipernokaidoyitau* are pinnately nerved throughout with 4–5 ascending secondary veins on each side. This characteristic is shared by other species belonging to the *Radula* clade, specifically subclade *Isophyllon* (Jaramillo et al. 2008). We compared P.nokaidoyitau with similar species that occur on the eastern slope of the northern Andes (Table 4).

**Table 4.** Comparison of *Pipernokaidoyitau* with related species that have leaves pinnately nerved throughout and occur in the eastern slope of the northern Andes.

Species	Prophylls length	Leaf shape	Leaf base shape	Leaf length	Infructescence length	Geographic distribution
***P.anonifolium* Kunth**	0.5–0.8 cm	elliptic	cuneate	(10)14–17 cm	3–3.5 cm	Amazonia
***P.hostmannianum* C. DC.**	1–2 cm	elliptic	rounded	12–20 cm	10–11 cm	Amazonia
***P.nokaidoyitau* W. Trujillo & M. A. Jaram.**	3–3.5 cm	ovate	rounded	21–26 cm	11–13 cm	Eastern slope of the northern Andes
***P.mastersianum* C. DC.**	0.4–0.8 cm	ovate	rounded	9–12 cm	4–9 cm	Amazonia

##### Specimens examined.

Colombia. Caquetá, municipio de Florencia: corregimiento El Caraño, vereda El Caraño, finca Las Brisas, 01°44'14.7"N, 75°40'35.3"W, 1116 m elev., 18 Oct 2013 [fl], *W. Trujillo et al. 3005* (COL); Corregimiento El Caraño, finca Las Brisas, 01°44'14.7"N, 75°40'35.3"W, 1116 m elev., 18 Oct 2013 [fl], *W. Trujillo et al. 3022* (COL); Municipio de Belén de los Andaquíes: río Pescado, Parque Natural Andaqui, sector sur, 01°36'31"N, 75°55'16"W, 950 m elev., 25 Jun 2013, *W. Trujillo et al*. 2791 (HUAZ). Cauca. Municipio de Piamonte, corregimiento de Miraflor, vereda La Florida, camino a la reserva La Cristalina, 01°04'59.6"N, 76°28'08.2"W, 1146 m elev., 06 Jan 2021 [fr], *E. Trujillo et al. 7249* (CUVC, JBB).

#### ﻿ Piper velae

Taxon classificationPlantaePiperalesPiperaceae

W. Trujillo-C & M. A. Jaram. sp. nov.

4BAD96EB-8763-5066-959F-55F6618FD24D

urn:lsid:ipni.org:names:77303989-1

**Figure 9:**
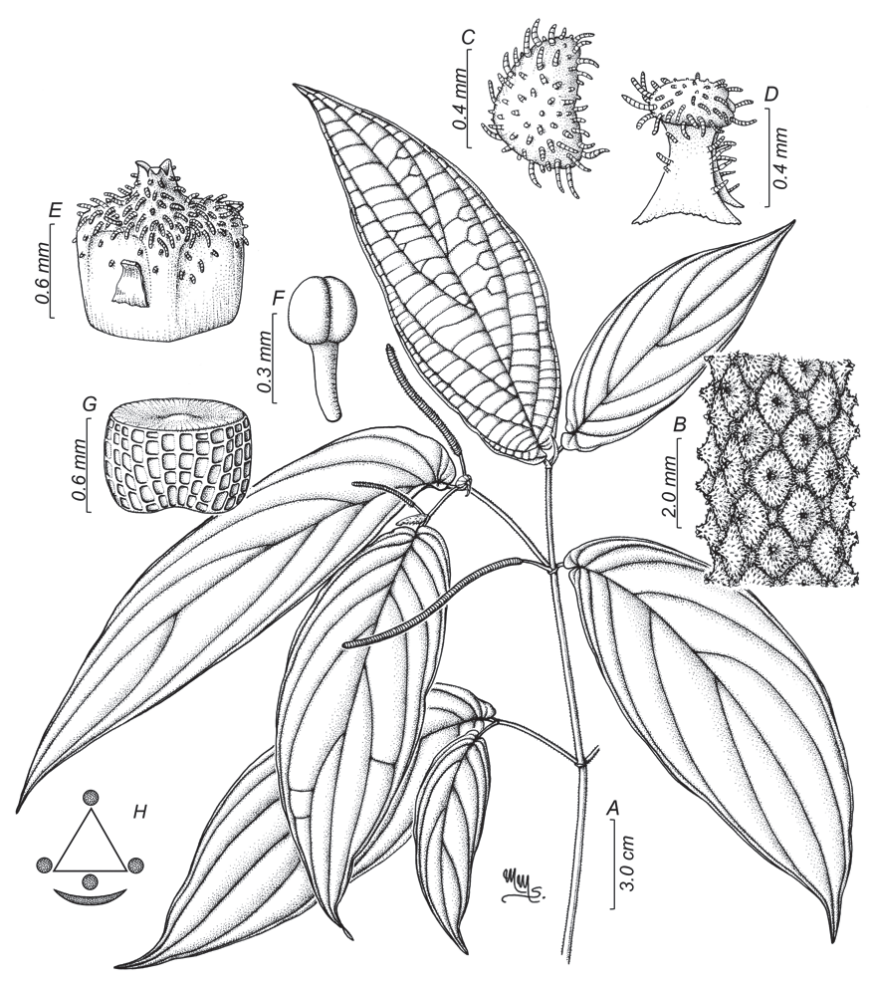
*Pipervelae***A** sympodial branch **B** magnified view of the infructescence **C** floral bract view from above **D** floral bract side view **E** fruit side view **F** stamen side view **G** Seed side view **H** floral diagram. Illustration by Marcela Morales based on *W Trujillo 905*

**Figure 10:**
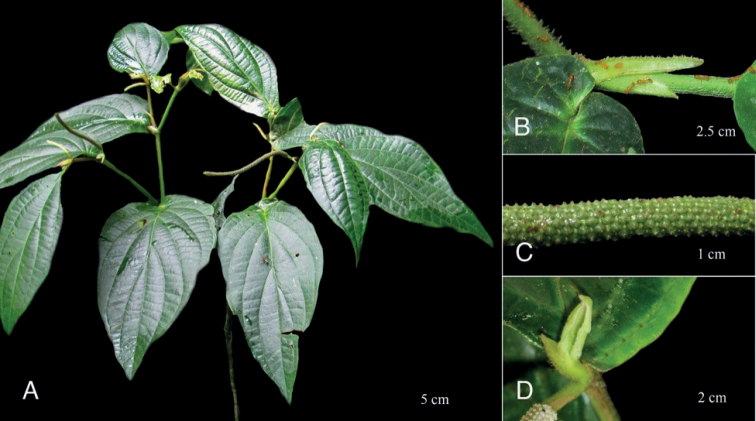
*Pipervelae***A** sympodial branch upper leaf surfaces and spike **B** magnified view of the leaf base and prophyll **C** magnified view of the spike **D** magnified view of the prophyll. Photos from *W. Trujillo 2039* by William Trujillo.

Figs 1 , 2 , 9 and 10

##### Type.

Colombia, Caquetá, Belen de los Andaquies, corredor resguardo La Cerinda, PNN Alto Fragua Indiguazi, etnia Embera Katio, 1°36'08.6"N, 75°51'49.1"W, 470 m elev., 03 Oct 2007, *W. Trujillo et al. 905* (Holotype COAH, Isotype HUAZ).

##### Diagnosis.

*Pipervelae* W. Trujillo & M. A. Jaram. can be distinguished from the related species *P.holdridgeanum* W.C. Burger by its elliptic leaves with cordulate leaf bases at all nodes, petioles 0.8–1.5 cm long, fruits cylindrical and pubescent vs. leaves cordate to elliptical with leaf bases that are rounded at fertile nodes and cordate at sterile nodes, petioles that are variable in size from 1–5 cm long, fruits rounded and glabrous in *P.holdridgeanum*. It can be separated from similar species *P.cornifolium*Kunth (1815 [1816]:52) because it has leaves pinnately nerved in the lower half of the blade and pubescent fruits vs. leaves pinnately nerved in the lower third of the blade and glabrous fruits in *P.cornifolium*.

##### Description.

***Shrub*** up to 1.5 m tall. Internodes 2–8.5 cm long, smooth, green, tomentulose, idioblasts not evident. ***Prophylls*** 1.2–2 cm long whitish, tomentulose, caducous. ***Petioles*** variable along all axes; on monopodial axes 1–1.5 cm long, vaginate to 3/4 of the length, smooth, tomentulose; on sympodial axes 0.8–1.2 cm long, vaginate at the base, smooth, tomentulose. ***Leaf-blades*** coriaceous, drying black, uniform in shape and size along all axes, 6–7(11) × 12–15(19) cm, elliptic, symmetric, base cordulate, basal extension asymmetrical; leaf blade smooth, tomentulose on the abaxial surface and glabrous adaxially, eciliate; pinnately nerved from the lower half, 4–5 ascending nerves on each side, festooned brochidodromous, with spacing decreasing and angle increasing towards the base, tertiary veins percurrent; apex acuminate. ***Inflorescences*** and infructescence a solitary spike, terminal, erect; peduncle 0.8–1.5 cm long, tomentulose, green; rachis in flower not seen, rachis in fruit 6–8.5 cm long, fruits densely grouped along the rachis. ***Floral bracts*** cucullate, triangular from above, 0.15–0.25 × 0.3–0.4 mm, glabrous on the adaxial surface, margin fimbriate, not forming bands around the spike. ***Flowers*** with four stamens, filaments 0.2–0.4 mm long, anthers 0.2–0.3 × 0.15–0.25 mm, longitudinally dehiscent, dithecous, shorter than filament, with connective not protruding, glabrate, idioblasts not evident, colour not seen. Stigmas 3, on a short style. ***Fruits*** cylindrical, laterally compressed, green when alive and black to brown when dry, 0.8–1.2 × 1–1.3 mm, pubescent on the tip, partially immersed in the rachis, with stigmas persistent, 0.07–0.12 mm long, on a short style, 0.1–0.3 mm long. ***Seeds*** 0.4–0.6 × 0.9–1.1 mm, rectangular, laterally compressed, obtuse, black.

##### Distribution and habitat.

*Pipervelae* occurs in the eastern slopes of the Andes, from 250–1,500 m in elevation, spreading from wet lowland to wet premontane forests. It occurs in the Colombian Departments of Caquetá, Meta, Cauca and Putumayo. In lowland forests, it occurs in dense *terra firme* forests. In premontane forests, it grows mostly on moderate slopes, sometimes occurring on steep slopes and rocky substrates. It is a shade-loving species, growing in the understorey and it is also found in forest gaps.

##### Phenology.

Flowering specimens were collected in February, April, May, June, July and October. Fruiting specimens in January, April, June, July, September, October and December.

##### Etymology.

*Pipervelae* is named in honour of Huber Fernando Vela, M.D., a social and environmental leader of Caquetá who was murdered in 2021. Dr Vela and sponsored *Piper* collections by WT during 2020. Huber Fernando was the leader of the Nature Reserve Romi Kumu, where 30 ha of forest were restored in 2020. The type specimen of *P.velae* occurs in the region that Dr Vela loved and helped conserve and restore.

##### Conservation status.

This species is known from 41 specimen collections representing 12 subpopulations. It occurs in 18 locations threatened by deforestation. The extent of occurrence (EOO = 40,810 km^2^, below the EOO to be considered Vulnerable, VU) and area of occupancy (AOO = 96 km^2^), suggest it is of Near Threatened [NT B1a+B2a].

##### Phylogenetic relationships.

*P.velae* is sister to *P.holdridgeanum* and these form a clade sister to *Macrostachys* (Jaramillo et al. 2008). *P.velae* and *P.holdridgeanum* have sheathing petioles to ¾ of their length and tightly-arranged flowers. The marked foliar dimorphism between leaves on sterile (monopodial) and fertile (sympodial) nodes distinctive of *P.holdridgeanum* have obscured its relationships (Callejas-Posada 2020). Here, we present phylogenetic evidence for its placement sister to *Macrostachys*. Both species, *P.velae* and *P.holdridgeanum* require further study to understand their morphological affinities.

##### Discussion.

*Pipervelae* can be confused with *P.cornifolium*, because of its cordulate leaf base; however, these taxa are distinguished, based on the leaf venation pattern and fruit pubescence (see Table 5). We also compare *P.velae* to closely-related *P.holdridgeanum*; further studies will help us corroborate this relationship and find morphological similarities.

**Table 5.** Comparison of *Pipervelae* with closely-related species *P.holdridgeanum* and morphological similar species *P.cornifolium*.

Species	Petiole length	Leaf shape	Leaf base	Secondary nerves branched	Fruit shape	Fruit pubescence	Geographic distribution
***P.cornifolium* Kunth**	0.8–1.2 cm	obovate-elliptic	cordulate	from the lower third	rounded	pubescent	Northern Andes
***P.holdridgeanum* W.C. Burger**	1–5 cm	cordate to elliptic	rounded to cordate	from the lower half	rounded	glabrous	Mesoamerica
***P.velae* W. Trujillo & M. A. Jaram.**	0.8–1.5 cm	elliptic	cordulate	from the lower half	cylindrical	pubescent	Eastern slope of the northern Andes

##### Specimen examined.

Colombia: **Caquetá**: Belén de los Andaquíes: Parque Bosque Microcuenca La Resaca, sendero Alto Sarabando, 800 m elev., 1°27'29"N, 75°53'1.8"W, 26 Oct 2010, *D. Cárdenas et al. 40791* (COAH); vereda Las Verdes, río Pescado, margen izq. Parque Natural Municipal Andakí, 700 m elev., 1°36'7.2"N, 75°54'10"W, 28 Oct 2010, *D. Cárdenas et al. 40896* (COAH); sector Paramillo, camino entre Acevedo - Belén de Andaquíes, 1400 m elev., 1°40'58"N, 75°54'21"W, 23 Jul 2011, *D. Cárdenas et al. 41848* (COAH); cerca del río Pescado, vereda Los Angeles, 1225 m elev., 1°34'49.7"N, 75°54'17"W, 9 Jul 2011, *L. Martinez 24* (COAH); río Pescado, Parque Natural Andakí, sector Sur, 950 m elev., 1°36'31"N, 75°55'16"W, 25 Jun 2013, *W. Trujillo et al. 2792* (COAH). Cartagena del Chairá: vereda Laguna del Chairá, 240 m elev., 1°15'23.9"N, 74°48'49.8"W, 28 Sep 2007, *W. Trujillo et al. 853* (COAH). Florencia: corregimiento El Caraño, vereda Alto Brasil, camino hacia la quebrada Yumal, 321 m elev., 1°39.543'N, 75°36.128'W, 22 Oct. 2012, *A. Jimenez et al. 4* (HUAZ); río Hacha, vereda El Caraño, finca Marsella, 500 m elev., 1°41'47"N, 75°37'16"W, 26 Jun 2010, *D. Cárdenas et al. 24884* (COAH, HUAZ); vereda El Paraíso, antigua vía Florencia-Neiva, zona de cordillera baja, 756 m elev., 1°45'7.4"N, 75°39'56.9"W, 20 Oct 2010, *D. Cárdenas et al. 40568* (COAH); vereda El Paraíso Bajo, vegetación de cordillera baja, 800 m elev., 1°45'0.2"N, 75°37'8.2"W, 22 Oct 2010, *D. Cárdenas et al. 40595* (COAH); Centro de investigaciones Macagual, 258 m elev., 1°29'59.8"N, 75°39'22.6"W, 13 Dec 2008, *W. Trujillo et al. 1214* (COAH); vereda El Caraño, 1116 m elev., 1°44'14.7"N, 75°40'35.3"W, 18 Oct 2013, *W. Trujillo et al. 3002* (COAH). La Montañita: vereda Los Morros, reserva Las Dalias, 300 m elev., 1°29'21.5"N, 75°24'17.6"W, *A. Meneses 15* (HUAZ); vereda Itarca, Reserva Natural Itarca, 340 m elev., 1°32'53"N, 75°28'20"W, 30 Oct 2010, *D. Cárdenas et al. 40956* (COAH); vereda Itarca, Reserva Natural Itarca, 330 m elev., 1°32'34.5"N, 75°28'19"W, 26 Apr 2011, *N. Castaño et al. 3142* (COAH); Reserva Las Dalias, 382 m elev., 1°29'21.6"N, 75°24'17.6"W, 41050, *W. Trujillo et al. 2086* (COAH). San Vicente del Caguán: inspección Guacamaya, vereda La Música, margen izquierda del río Caguán, 600 m elev., 2°20'46"N, 74°54'12.4"W, 12 Jul 2015, *D. Cárdenas et al. 44401* (COAH). **Cauca**: Piamonte: La Libertad, 700 m elev., 1°6'41.78"N, 76°28'46.5"W, 22 Feb 2010, *W. Trujillo et al. 1271* (COAH). Santa Rosa: Inspección de Santa Marta, vereda Diamante Alto, 1150 m elev., 1°14'N, 76°36'W, 22 Jun 2002, *B. Ramírez 16061* (COAH). **Meta**: San Juan de Arama: sector del chorro Santo Domingo, Parque Nacional Natural Sierra de La Macarena, 520 m elev., 3°15'23"N, 73°57'50"W, 27 Oct 2019, *D. Cárdenas et al. 52002* (COAH); vereda Monserrate Bajo, finca El Paraíso, cerca al caño Las Ninfas, 590 m elev., 3°20'33.75"N, 73°57'3.06"W, 4 Apr 2004, *L. Carvajal et al. 173* (COAH). **Putumayo**: Mocoa: vereda Medio Afán, camino Serranía El Churumbelo, sector Nororiental, 900 m elev., 1°10'39"N, 76°38'47"W, 4 Oct 2000, *D. Cárdenas et al. 12221* (COAH); vereda San José del Pepino, 1°5'40.17"N, 76°37'49.05"W, 21 Jun 1997, *R. López et al. 2514* (COAH); vereda San José del Pepino, 1°5'40.17"N, 76°37'49.05"W, 21 Jun 1997, *R. López et al. 2610* (COAH). Orito: Santuario de Flora y Plantas Medicinales Ingui-Ande, vereda Líbano, sector de don Reinaldo, 900 m elev., 0°41.62'N, 77°3.789'W, 1 Oct 2015, *D. Cárdenas et al. 45416* (COAH); Santuario de Flora y Plantas Medicinales Ingui-Ande, 1004 m elev., 0°41.627'N, 77°3.801'W, 27 Sep 2015, *D. Cárdenas et al. 45454* (COAH); vereda El Líbano, 832 m elev., 0°38'15"N, 77°4'17.9"W, *D. Cárdenas et al. 51291* (COAH).

## Supplementary Material

XML Treatment for Piper hoyoscardozii

XML Treatment for Piper indiwasii

XML Treatment for Piper nokaidoyitau

XML Treatment for Piper velae
